# Chronic Maternal Depressive Symptoms Are Associated With Reduced Socio-Emotional Development in Children at 2 Years of Age: Analysis of Data From an Intervention Cohort in Rural Pakistan

**DOI:** 10.3389/fpsyt.2019.00859

**Published:** 2019-11-20

**Authors:** Clariana V. R. De Oliveira, Muneera Rasheed, Aisha K. Yousafzai

**Affiliations:** ^1^Department of Global Health and Population, Harvard T.H. Chan School of Public Health, Boston, MA, United States; ^2^Department of Paediatrics and Child Health, Aga Khan University, Karachi, Pakistan

**Keywords:** maternal depression, chronicity, early child development, low- and middle-income countries, cognition, language, motor skills, socio-emotional development

## Abstract

**Background:** Maternal depression affects a high proportion of women during the antenatal and postnatal period in low- and middle-income countries. While maternal depression is recognized as a significant risk for poor early child development that warrants interventions, the effects of chronic maternal depression on children’s development are less understood.

**Objective:** To determine the association of chronicity of maternal depressive symptoms and early child development in a rural population in southern Pakistan.

**Materials and Methods:** This study employs data from the “Pakistan Early Child Development Scale-Up Trial,” a randomized controlled study that evaluated the integration of responsive stimulation and nutrition interventions in a community health service. In the present analysis, linear regression was used to test the effects of chronicity of high maternal depressive symptoms on children’s early development (n = 1205 mother-infant dyads). Children’s development was assessed using the Bayley Scales of Infant and Toddler Development at 24 months of age. Maternal depressive symptoms were assessed at baseline and every 6 months using the Self-Reporting Questionnaire.

**Results:** No significant associations were observed between chronic maternal depressive symptoms and child cognitive, language, or motor development after adjusting for parental characteristics, the caregiving environment and socioeconomic variables. A negative significant association between chronicity of high maternal depressive symptoms and child socio-emotional development (β coefficient −2.57, 95% CI: −5.14; −0.04) was observed after adjusting for the selected variables.

**Conclusions:** The results suggest that interventions designed to promote early child development should also integrate repeat screening for depression and longer-term psychosocial support for mothers.

## Introduction

Maternal depression affects 15.6% and 19.8% of women during antenatal and postnatal periods respectively in low- and middle-income countries (LMIC) (1). Low socioeconomic status (SES), inadequate support for mothers, stress, exposure to violence, and natural disasters may increase risks for depression, which might explain some differences in the higher reported prevalence of maternal depression in LMIC compared with high-income countries (1). Like many other LMIC, Pakistan has high reported rates of antenatal depressive symptoms ranging from 16.8% to 55.9% (2, 3) and of postnatal depressive symptoms ranging from 28.8% to 36% (4, 5). Several studies in Pakistan have investigated depression following child birth (6). In one study, chronicity of depression was assessed and found half of Pakistani mothers who were depressed in the third trimester of pregnancy continued to be depressed one year after giving birth (7).

Maternal depressive symptoms (e.g., distress, depressed mood, loss of interest, and enjoyment) are associated with poor child health, nutrition, and growth outcomes, which may be the result of poorer maternal sensitivity and responsiveness to her child’s needs as a result of depression (1, 8, and 9). A systematic review and meta-analyses of the effect of maternal depression or depressive symptoms on child growth in LMIC reported that maternal depression was associated with early childhood underweight and stunting compared with children whose mothers were not presenting depression or depressive symptoms (10). Specifically, in Pakistan, studies have reported an increased risk of underweight (11) and diarrhea episodes (12), and an increased risk of early cessation of exclusive breastfeeding (13) in infants of depressed mothers compared with infants of psychologically well mothers.

With respect to child development outcomes, a meta-analysis of 14 studies from 10 countries reported an association between maternal depressive symptoms and lower cognitive scores in early infancy (14). In a study from the United Kingdom, children whose mothers had high depressive symptoms during the early postpartum period had higher levels of dysregulated infant behavior and lower cognitive scores compared with children with non-symptomatic mothers (15). In Canada, a study showed that maternal depressive symptoms increased risks of children having low receptive vocabulary and displaying inattention or physical aggression at 4 to 5 years of age compared to children whose mothers had no depressive symptoms (16). Fewer studies from LMIC have investigated the relationship between maternal depression and child development. However, one study from Vietnam reported higher symptoms of common mental disorders (including depression and anxiety) in mothers during the third trimester of pregnancy were significantly associated with low scores for children’s motor development (17).

A key factor believed to moderate the effects of maternal depression on child outcomes is the chronicity of depressive symptoms (18). At present, only a few studies from high-income countries have examined the effects of the chronicity of maternal depressive symptoms on children’s health and development outcomes. The limited evidence suggests that young children exposed to more severe and chronic maternal depressive symptoms have greater health and development problems. For example, Prenoveau and colleagues found that chronic maternal depressive symptoms during the first two years postpartum (assessed at 3, 6, 10, 14, and 24 months) were related to poorer behavior and emotional negativity in children at 24 months of age (18). Similarly, Cents and colleagues modeled trajectories of maternal depressive symptoms during pregnancy through the first 36 months of life and identified that children of mothers with higher depressive symptoms had significantly more behavior problems than children of mothers with lower trajectories of depressive symptoms (19). Brennan and colleagues observed the severity and chronicity of maternal depressive symptoms assessed during pregnancy and again when the child was 6 months old and 5 years old was significantly associated with child behavior problems (20). Cornish and colleagues reported that chronic maternal depression (assessed at 4, 12, and 15 months postpartum) was associated with lower infant cognitive and psychomotor development at 15 months of age, while brief depressive symptoms did not signiﬁcantly impact the child’s performance (21).

There is a need to evaluate the impact of chronic maternal depressive symptoms on child development outcomes in populations with greater risks of exposure to depressive symptoms. A number of other risk factors (e.g., fewer early learning opportunities) may further compound early development in such contexts. Understanding the contribution of these risks and their impacts on the developing child are critical to inform targeted intervention strategies to support parenting (22). The Pakistan Early Child Development Scale-Up Trial (PEDS-trial) was a pragmatic community-based cluster randomized factorial effectiveness trial evaluating the effects of early responsive stimulation intervention (with or without nutrition intervention) on child development in the first 2 years of life (23). The study found responsive stimulation (with or without nutrition) signiﬁcantly beneﬁtted children’s cognitive, language, and motor development as well as parenting knowledge and practices, and caregiver-child interactions (23, 24).

With respect to maternal depressive symptoms, the PEDS trial found that 29% of mothers had high depressive symptoms and 12% expressed suicide ideation at the time of enrolment (<2.5 months postpartum) (24). The combined responsive stimulation and nutrition intervention had a modest signiﬁcant effect on decreasing maternal depressive symptoms over time. However, the impact of chronicity of maternal depressive symptoms on children’s development has not been reported. The objective of this study was to evaluate whether chronicity of maternal depressive symptoms affects child development at 24 months of age.

## Materials and Methods

### Study Design, Setting, and Population

This analysis employs data from the “Pakistan Early Child Development Scale Up (PEDS)” trial implemented in district in Sindh province, Pakistan. This is a predominantly rural agricultural districts. Families are exposed to high levels of adversity. On average, the monthly household income was Pakistani Rupees 9826.60 (US$ 62.37 based on current exchange rates). One-third of households were food insecure. With respect to parental education, 68% of mother and 31% of fathers were illiterate. Childhood malnutrition was prevalent with 43% of children underweight and 61% of children stunted (short stature) by 24 months of age.

The PEDS trial was a cluster-randomized factorial effectiveness trial (23). A total of 1489 mother–infant dyads were enrolled at birth and 1391 mother-infant dyads were followed for 2 years (23). A birth cohort was recruited with their mothers from the study clusters. Births were identified by an independent surveillance team. Every infant born in the study area between April 1^st^ 2009 and March 31^st^, 2010 was eligible for enrolment. Inclusion criteria for enrolment were infants <2.5 months of age without signs of disability who were born and living in the study cluster. Ethical approval was granted by the Ethics Review Committee of the Aga Khan University. The PEDS trial was registered at www.clinicaltrials.gov (NCT00715936).

This analytical sample included 1,205 mother-infants dyads. Criteria for inclusion were: (1) a completed child development assessment at 24 months; (2) the primary caregiver was the child’s biological mother; and (3) mothers had an assessment for depressive symptoms at all five-time points (baseline, 6, 12, 18, and 24 months). The primary outcomes in this analyses was children’s cognitive, language, motor, and socio-emotional development at 24 months of age.

### Outcome Assessment and Covariates

#### Child Development

The primary outcome of interest was early child development (ECD) measured by the Bayley Scales of Infant and Toddler Development, Third Edition (Bayley-III) when children were 24 months old. A trained team of assessors collected data on children’s cognitive, language (receptive and expressive), motor (fine and gross), and socio-emotional development. Composite scores were reported and a higher score denotes better development. Inter-observer agreement was high for all domains of development (cognitive development: N = 84, R = 0.99, p < 0.0001; receptive communication: N = 83, R = 0.97, p = 0.01; expressive communication: N = 84, R = 0.97, p = 0.001; social-emotional: N = 81, R = 0.99, p < 0.001; fine motor development: N = 83, R = 0.97, p < 0.0001; gross motor: development N = 83, R = 0.95, p < 0.0001) (23).

#### Self-Report Questionnaire-20 (SRQ-20)

Maternal depressive symptoms were assessed using the Self-Reporting Questionnaire (SRQ-20) (25). The SRQ-20 is a screening tool designed to measure neurotic symptoms and includes symptoms of anxiety as well as depressive symptoms or psychological distress. The SRQ-20 is not a diagnostic instrument; however, it has been used to screen for risk of clinical depression. The SRQ-20 comprises 20 questions each scored either 0 or 1 to indicate whether an individual has not experienced the symptoms or has experienced the symptom in the past month. A higher score denotes an increasing number of depressive symptoms. In Pakistan, the validity and reliability for postnatal depression using an adapted version of the SRQ-20 has been reported, and a score ≥9 indicates risk of postnatal depression (26, 27). The validation and reliability work was conducted in a population in Punjab Pakistan, in a peri-urban population. The current population in Sindh shares common disadvantages and cultural similarities. Additionally, the language translation from Urdu to Sindhi was reviewed to ensure constructs retained meaning and an independent back translation was completed for quality assurance (24).

In this study, the SRQ-20 was administered by interview in a private space to mothers at five time points at baseline and when children were 6, 12, 18, and 24 months old. Inter-observer agreement for the SRQ-20 administration was high (N = 51, R = 0.99, 0.001) (24). Chronicity was defined with respect to high maternal depressive symptoms when a mother had two consecutive scores on the SRQ-20 of ≥ 9 (see **Table 1**).

**Table 1:** Categories to describe chronicity of maternal depressive symptoms.

Category	Definition
*No high depressive symptoms reported at any time point*	Women with a SRQ-20 score <9 at each time point.
*High depressive symptoms reported at a single time point*	Women with a SRQ-20 score ≥9 for a single time point only.
*High episodic depressive symptoms*	Women with a SRQ-20 score ≥9 for 2 or more time points, but not continuously.
*Chronicity of depressive symptoms*	Women with a SRQ-20 score ≥9 for 2 or more consecutive time points.

#### Child Growth

Deficits in development are often seen in early childhood growth (28). Therefore, data on children’s growth status were collected following standard protocols (29). Height was measured with a ShorrBoard^©^ to the nearest 0.1 cm, and weight was measured with a Seca877 Digital Flat Scale with Mother/Child Function^©^ to the nearest 0.1 kg. The technical error of measurement for height was 1.86%, (R = 0.99); and for weight was 0.71% (R = 0.99). Weight-for-age (WAZ) and height-for-age (HAZ) at 24 months old were reported in accordance with the World Health Organization growth standards. A score of ≤−2 denotes moderate-to-severe undernutrition.

#### Observation of Mother and Child Interaction (OMCI)

Quality of caregiver-child interactions is associated with benefits to children’s development. Assessment of mother-child interaction was collected using the Observation of Mother and Child Interaction (OMCI) when children were 12 months old (30). The primary caregiver (typically the mother) was instructed to play with her child using a picture book while a trained observer rated a live 5-min interaction and scored frequency of behaviours. The tool comprises 19 items covering maternal affect, maternal touch, maternal verbalization, sensitivity, contingent responses, scaffolding, language stimulation, focus, child affect, child focus, child’s communication efforts, and mutual enjoyment. A high score denotes greater frequency of behaviours associated with good quality interaction. Inter-observer agreement was moderate-to-high (N = 81, R = 0.82, p < 0.001) (24).

#### Home Observation for Measurement of the Environment (HOME) Inventory

The Home Observation for Measurement of the Environment (HOME Inventory) is the most widely recognized and commonly used instrument to evaluate the quality of the caregiving and learning environment for young children (31). High scores on the HOME Inventory (the Infant and Toddler version) are associated with benefits to children’s development (32). The HOME Inventory employs observation and interview methods to assess the following caregiving domains: responsivity, acceptance, organization, learning materials, involvement, and variety. A higher score denotes greater quality of the environment that supports early development and learning. In this analysis, we used HOME Inventory data collected when children were 18 months old. Inter-observer agreement was high (N = 96, R = 0.99, p < 0.001) (21).

#### Household Data

Data on household SES were collected from each enrolled household at baseline. A composite wealth index measure of a household’s cumulative living standard was constructed using household asset data including ownership of a number of consumer items ranging from a television to a bicycle or car, as well as the source of drinking water, sanitation facilities, type of material used for flooring, and household income (23). Several studies have reported a significant association between low SES and poor outcomes in the child development (33).

Food security data were also collected from each household using a standardized questionnaire that asks about access and sufficiency of food among household members in the past 4-weeks. Food security describes a situation when the family has physical and economic access to sufficient, safe, and nutritious food to meet their dietary needs for an active and healthy life as opposed to food insecurity when there is a situation of limited or uncertain availability of nutritionally adequate and safe foods (34).

Data on the number of siblings and birth order were used for this analysis. Siblings can promote an environment of support for child development, but depending on the order of birth may also influence the amount of attention that caregivers might provide to a child (35). Parental education data were collected on both mothers and fathers. The greater the number of years of formal education parents receive is positively associated with child development outcomes (22).

### Statistical Analysis

**Figure 1:**
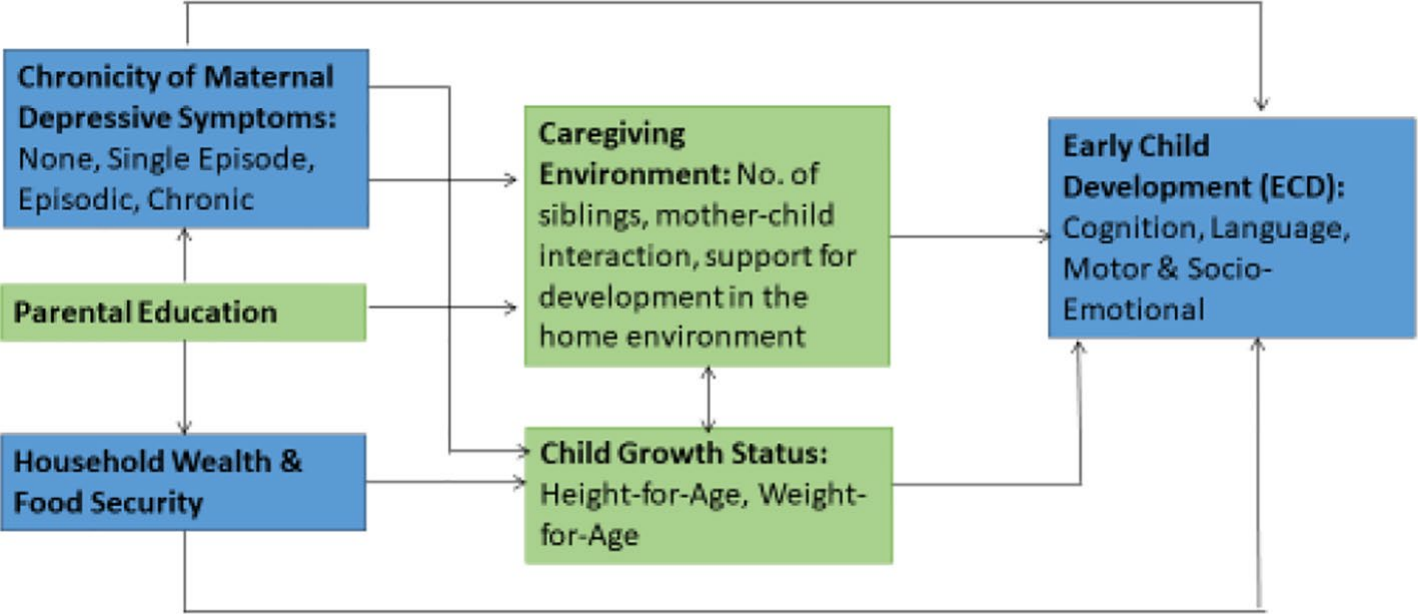
Hypothesized pathways between maternal depression and child development. Adapted from Grantham-McGregor et al. (28).

**Figure 1** shows the theoretical pathways of interest for the analysis. Stata 14.1 (StataCorp, College Station, Texas, USA) was used to perform the analysis. The dependent variables were the Bayley-III composite scores for children’s cognition, language, motor and socio-emotional development. Chronicity of maternal depressive symptoms (i.e., no high depressive symptoms, single episode of high depressive symptoms, episodic high depressive symptoms, and chronicity of high depressive symptoms) was the exposure variable. For categorical outcomes, we used *t*-tests, one-way ANOVA, and χ^2^ tests to visualize the relationship between exposures and continuous outcomes. The Shapiro-Wilk test, scatter-plots, and comparisons between mean and median values were used to assess the normality of continuous variables.

Four models for each child development outcome were analyzed using a step-wise approach. In the first model, exposure was tested adjusting for child characteristics (i.e., gender, height for age, weight for age, and birth order). In the second model, exposure was tested adjusting for parental education. In the third model, exposure was tested adjusting for variables that represented the child’s caregiving environment (i.e., number of siblings, caregiving environment, and mother-child interactions). In the fourth model, exposure was tested adjusting for household SES and food security. Sixteen multiple linear regression models were used to estimate the effect of chronicity of maternal depressive symptoms on child development domains at 24 months of age. Models were controlled for clustering effects and intervention exposure. The missing data were included in the multiple models by creating missing-value categories. The fit of the model was ascertained by an examination of residuals, which did not show any potential harmful effects. Collinearity was examined by the correlation matrix.

## Results

A total of 1205 mother-infant dyads were analyzed. The characteristics of the participants are presented in **Table 2**. We observed that 35.5% of mothers had chronicity of high depressive symptoms, 11.7% had experienced episodic high depressive symptoms, and 22.3% had experienced a single episode of high depressive symptoms. A total of 69.5% of Pakistani mothers experienced high depressive symptoms during the first 24 months of her child’s life.

**Table 2:** Characteristics of the study population.

Variables	N	Value
		Mean (SD)
Bayley-III score of cognitive at 24 months^1^	1.205	78.48 (14.65)
Bayley-III score of language at 24 months^1^	1.205	82.95 (13.55)
Bayley-III score of social-emotional at 24 months^1^	1.205	94.00 (0.25)
Bayley-III score of motor at 24 months^1^	1.205	89.07 (16.99)
Height-for-age^1^ (24m)	1.197	−0.84 (1.16)
Weight-for-age^1^ (24m)	1.198	−0.81 (0.95)
Birth order^1^ (Baseline)	1.205	3.49 (2.30)
Number of siblings^1^ (Baseline)	1.205	4.18 (2.26)
HOME quality^1^ (18m)	1.205	30.92 (5.41)
OMCI^1^ (12m)	1.203	30.0 (8.61)
Wealth (Baseline)	1.199	0.01 (0.99)
		%
Chronicity of Maternal Depression Symptoms *^2^*		
No high depressive symptoms at any time point	367	30.5
Single high depressive symptoms	269	22.3
Episodic high depressive symptoms	141	11.7
Chronicity	428	35.5
Gender^2^ (Baseline)		
Female	554	46.0
Male	651	54.0
Maternal education^2^ (Baseline)		
No education	170	14.1
Primary and above	1.035	85.9
Father education^2^(Baseline)		
No education	688	57.1
Primary and above	517	42.9
Food insecurity^2^ (Baseline)	1.205	
Secure	771	63.98
Insecure	434	36.02

Continuous variable variable^1^; categorical variable^2^; HOME = Home Observation for Measurement of the Environment; OMCI = Observation of Mother and Child Interaction; Chronicity of Maternal Depression Symptoms = No high depressive symptoms at any time point refers to women with a SRQ-20 score <9 at each time point; high depressive symptoms reported at a single time point refers to women with a SRQ-20 score ≥9 for a single time point only; high episodic depressive symptoms refers to women with a SRQ-20 score ≥9 at 2 or more time points, but not continuously; and chronicity refers to women with a SRQ-20 score ≥9 at 2 or more consecutive time points.

In the multivariate analysis for cognitive development, adjusting for child characteristics, the results for the first model found that episodic and chronicity of high depressive symptoms were associated with poorer child cognitive development with a decrease in the β coefficient (episodic β coefficient −4.37, 95% CI: −7.15; −1.59; chronic β coefficient −2.73, 95% CI: −4.74; −0.73). This association remained in model 2 after adjusting for parental education only for the episodic high depressive symptoms, but not in models 3 and 4 (episodic β coefficient −2.02, 95% CI: −4.58; 0.52; chronicity β coefficient −1.02, 95% CI: −2.89; 0.85) after adjusting for the caregiving environment and household variables. In the final model we observed that only child HAZ, HOME, OMCI, and SES were significantly associated with child cognitive outcome (see **Table 3**).

**Table 3:** Multivariate analysis of maternal depressive symptoms and child cognitive development.

Variables	Model 1		Model 2		Model 3		Model 4	
	*β* ^a^	CI^b^	*β* ^a^	CI^b^	*β* ^a^	CI^b^	*β* ^a^	CI^b^
Chronicity of Maternal Depression Symptoms								
No high depressive symptoms at any time point	*1*		*1*		*1*		*1*	
Single high depressive symptoms	−1.44	−3.69; 0.79	−1.13	−3.35; 1.09	−.59	−2.92; 1.19	−0.59	−2.61; 1.43
Episodic high depressive symptoms	−4.37	−7.15; −1.59*	−3.79	−6.55; −1.03*	−1.87	−4.44; 0.68	−2.02	−4.58; 0.52
Chronicity	−2.73	−4.74; −0.73*	−1.78	−3.80; 0.22	−1.08	−2.95; 0.77	−1.02	−2.89; 0.85
Male	0.52	−1.09; 2.14	0.67	−0.93; 2.27	0.75	−0.72; 2.24	0.72	−0.73; 2.18
Height-for-age (24 m)	2.65	1.58; 3.72*	2.29	1.23; 3.36*	1.60	0.66; 2.64*	1.60	0.61; 2.59
Weight-for-age (24 m)	−0.27	−1.57; 1.01	−0.22	−1.51; 1.05	−0.20	−1.39; 0.98	−0.19	−0.73; 2.18
Birth order	−0.61	−0.97; −0.26*	−0.45	−0.80; −0.09*	−0.08	−1.12; 1.11	−0.09	−1.20; 1.00
Maternal education			−2.30	−3.48; −1.11*	−0.84	−1.96; 0.27	−0.76	−3.05; 1.51
Father education			−1.30	−2.26; −0.33*	−0.50	−1.40; 0.39	−0.02	−1.65; 1.61
Number of siblings					−0.27	−1.41; 0.87	−0.21	−1.34; 0.90
HOME quality (18 m)					0.74	0.59; 0.90*	0.62	0.47; 0.78*
OMCI (12 m)					0.33	0.24; 0.42*	0.26	0.17; 0.36*
*Food insecurity*							0.57	−1.11; 2.26
Wealth							1.96	1.07; 2.86*
*N*	1.205		1.205		1.205		1.205	
*R* *^2^*-adjusted	.05		.08		.20		.22	

^a^β-coefficient linear regression; ^b^Confidence Interval; *P-value <0.05; HOME = Home Observation for Measurement of the Environment; OMCI = Observation of Mother and Child Interaction; Chronicity of Maternal Depression Symptoms = No high depressive symptoms at any time point refers to women with a SRQ-20 score <9 at each time point; high depressive symptoms reported at a single time point refers to women with a SRQ-20 score ≥9 for a single time point only; high episodic depressive symptoms refers to women with a SRQ-20 score ≥9 at 2 or more time points, but not continuously; and chronicity refers to women with a SRQ-20 score ≥9 at 2 or more consecutive time points.

For children’s language outcome, the multivariable analysis in model 1, adjusting for child characteristics, showed that episodic and chronicity of high depressive symptoms were associated with a 2.5 and a 3.1 units decrease in the scale of language development (β coefficient −2.52, 95% CI: −5.10; −0.05, β coefficient −3.12, 95% CI: −4.98; −1.26). In model 2, after adjusting for parental education, this association was no longer significant (β coefficient −2.00, 95% CI: −4.56, −0.54; β coefficient −2.26, 95% CI: −4.12; 0.39). In the final model, only child HAZ, HOME, OMCI, and SES were significantly associated with children’s language development (see **Table 4**).

**Table 4:** Multivariate analysis of maternal depressive symptoms and child language development.

Variables	Model 1		Model 2		Model 3		Model 4	
	*β* ^a^	CI^b^	*β* ^a^	CI^b^	*β* ^a^	CI^b^	*β* ^a^	CI^b^
Chronicity of Maternal Depression Symptoms								
No high depressive symptoms at any time point	*1*		*1*		*1*		*1*	
Single high depressive symptoms	−1.66	−3.74; 0.41	−1.37	−3.43; 0.68	−1.15	−3.05; 0.75	−0.69	−2.55; 1.16
Episodic high depressive symptoms	−2.52	−5.10; −0.05*	−2.00	−4.56; 0.54	−0.27	−2.65; 2.09	0.11	−2.23; 2.46
Chronicity	−3.12	−4.98; −1.26*	−2.26	−4.12; 0.39	−1.64	−3.37; 0.08	−1.17	−2.90; 0.54
Male	−0.51	−2.02; 0.98	−0.38	−1.87; 1.09	−0.33	−1.71; 1.04	−0.21	−1.55; 1.12
Height-for-age (24 m)	2.26	1.27; 3.25*	1.93	0.94; 2.92*	1.35	0.43; 2.27*	1.30	0.40; 2.21*
Weight-for-age (24 m)	0.12	−1.07; 1.32	0.17	−1.01; 1.35	0.16	−0.93; 1.26	0.11	−1.96; 1.19
Birth order	−0.51	−0.84; −0.18*	−0.36	−0.69; −0.03*	−0.34	−1.38; 0.68	−0.48	−1.49; 0.52
Maternal education			−2.14	−3.24; −1.05*	−0.84	−1.88; 0.18	0.16	−1.93; 2.27
Father education			−1.12	−2.00; −0.23*	−0.38	−1.22; 0.44	0.27	−1.22; 1.78
Number of siblings					0.15	−0.90; 1.21	0.26	−0.76; 1.30
HOME quality (18 m)					0.65	0.51; 0.80*	0.53	0.39; 0.67*
OMCI (12 m)					0.33	0.24; 0.41*	0.24	0.15; 0.32*
Food insecurity							0.06	−1.56; 1.54
Wealth							2.17	1.34; 2.99*
*N*	1.205		1.205		1.205		1.205	
*R* *^2^*-adjusted	.05		.07		.21		.24	

^a^β-coefficient linear regression; ^b^Confidence Interval; *P-value <0.05; HOME = Home Observation for Measurement of the Environment; OMCI = Observation of Mother and Child Interaction; Chronicity of Maternal Depression Symptoms = No high depressive symptoms at any time point refers to women with a SRQ-20 score <9 at each time point; high depressive symptoms reported at a single time point refers to women with a SRQ-20 score ≥9 for a single time point only; high episodic depressive symptoms refers to women with a SRQ-20 score ≥9 at 2 or more time points, but not continuously; and chronicity refers to women with a SRQ-20 score ≥9 at 2 or more consecutive time points.

Child motor development was associated with episodic and chronicity of high depressive symptoms in model 1 adjusting for child characteristics with decreases in the scale of 4.3 and 3.4 units respectively. This association remained significant only for episodic high maternal depressive symptoms in model 2, after adjusting for parental education. No significant associations were found in models 3 and 4 after adjusting for the caregiving environment and household variables respectively. In the final model, only child HAZ, HOME, OMCI, and SES were significantly associated with motor development (see **Table 5**).

**Table 5:** Multivariate analysis of maternal depressive symptoms and child motor development.

Variables	Model 1		Model 2		Model 3		Model 4	
	*β* ^a^	CI^b^	*β* ^a^	CI^b^	*β* ^a^	CI^b^	*β* ^a^	CI^b^
Chronicity of Maternal Depression Symptoms								
No high depressive symptoms at any time point	*1*		*1*		*1*		*1*	
Single high depressive symptoms	−1.59	−4.19; 0.99	−1.34	−3.92; 1.23	−0.13	−3.55; 1.28	−0.61	−3.00; 1.77
Episodic high depressive symptoms	−4.29	−7.50; −1.07*	−3.71	−6.91; −0.50	−1.79	−4.81; 1.22	−1.58	−4.59; 1.42
Chronicity	−3.45	−5.77; −1.13*	−2.55	−4.49; 0.22	−1.90	−4.10; 0.28	−1.42	−3.63; 0.78
Male	1.15	−0.71; 3.03	1.31	−0.54; 3.17	1.36	−0.38; 3.11	1.40	−0.32; 3.12
Height-for-age (24 m)	3.32	2.08; 4.55*	2.99	1.76; 4.26*	2.37	1.20; 3.54*	2.18	1.01; 3.34*
Weight-for-age (24 m)	−0.40	−1.89; 1.09	−0.37	−1.86; 1.11	−0.38	−1.78; 1.00	−0.36	−1.74; 1.01
Birth order	−0.70	−0.94; −0.04*	−0.55	−0.96; −0.14*	−1.08	−2.40; 0.23	−1.20	−2.49; 0.09
Maternal education (b)			−1.72	−3.10; −0.34*	−0.29	−1.60; 1.02	0.52	−2.16; 3.22
Father education (b)			−1.63	−2.74; −0.51*	−0.77	−1.83; 0.28	−0.09	−1.82; 2.02
Number of siblings (b)					0.75	−0.58; 2.10	0.85	−0.47; 2.17
HOME quality (18 m)					0.74	0.56; 0.92*	0.59	0.41; 0.77*
OMCI (12 m)					0.37	0.26; 0.48*	0.30	0.18; 0.41*
Food insecurity (b)							−0.13	−2.12; 1.85
Wealth							2.60	1.55; 3.66*
*N*	1.205		1.205		1.205		1.205	
*R* *^2^*-adjusted	.06		.07		.18		.21	

^a^β-coefficient linear regression; ^b^Confidence Interval; *P-value <0.05; HOME = Home Observation for Measurement of the Environment; OMCI = Observation of Mother and Child Interaction; Chronicity of Maternal Depression Symptoms = No high depressive symptoms at any time point refers to women with a SRQ-20 score <9 at each time point; high depressive symptoms reported at a single time point refers to women with a SRQ-20 score ≥9 for a single time point only; high episodic depressive symptoms refers to women with a SRQ-20 score ≥9 at 2 or more time points, but not continuously; and chronicity refers to women with a SRQ-20 score ≥9 at 2 or more consecutive time points.

There was a significant negative association between chronicity of high maternal depressive symptoms and child socio-emotional development in all four models tested (β coefficient −2.57, 95% CI: −5.21; −0.04) (see **Table 6**). The HOME Inventory and OMCI remained significant independent variables associated with socio-emotional development.

**Table 6:** Multivariate analysis of maternal depressive symptoms and child socio-emotional development.

Variables	Model 1		Model 2		Model 3		Model 4	
	*β* ^a^	CI^b^	*β* ^a^	CI^b^	*β* ^a^	CI^b^	*β* ^a^	CI^b^
Chronicity of Maternal Depression Symptoms								
No high depressive symptoms at any time point	*1*		*1*		*1*		*1*	
Single high depressive symptoms	−1.33	−4.17; 1.51	−1.11	−3.95; 1.72	−0.94	−3.72; 1.83	−0.93	−3.72; 1.84
Episodic high depressive symptoms	−3.83	−7.36; −0.31*	−3.41	−6.94; 0.10	−2.08	−5.56; 1.38	−1.85	−5.36; 1.65
Chronicity	−3.82	−6.36; −1.28*	−3.14	−5.71; −0.57*	−2.63	−5.15; −0.10	−2.57	−5.14; −0.04*
Male	−0.03	−2.05; 2.04	0.10	−1.94; 2.15	0.22	−1.78; 2.23	0.32	−1.68; 2.33
Height-for-age (24 m)	1.69	0.34; 3.05*	1.44	0.08; 2.80*	1.01	−0.33; 2.35	1.05	−0.30; 2.40
Weight-for-age (24 m)	0.68	−0.95; 2.32	0.71	−0.92; 2.35	0.75	−0.85; 2.36	0.56	−1.04; 2.18
Birth order	−0.49	−0.94; −0.04*	−0.37	−0.83; 0.07	−0.47	−1.99; 1.03	−0.52	−2.03; 0.99
Maternal education			−1.55	−3.06; −0.03*	−0.52	−2.03; 0.98	−0.18	−2.95; 3.33
Father education			−1.02	−2.24; −0.20*	−0.46	−1.68; 0.74	−0.81	−3.05; 1.42
Number of siblings					0.25	−1.29; 1.80	0.28	−1.25; 1.83
HOME quality (18 m)					0.56	0.35; 0.76*	0.60	0.39; 0.82*
OMCI (12 m)					0.20	0.07; 0.32*	0.21	0.08; 0.34*
Food insecurity							0.72	−1.59; 3.04
Wealth							0.21	−1.01; 1.45
*N*	1.205		1.205		1.205		1.205	
*R* *^2^*-adjusted	.03		.04		.08		.08	

^a^β-coefficient linear regression; ^b^Confidence Interval; *P-value <0.05; HOME = Home Observation for Measurement of the Environment; OMCI = Observation of Mother and Child Interaction; Chronicity of Maternal Depression Symptoms = No high depressive symptoms at any time point refers to women with a SRQ-20 score <9 at each time point; high depressive symptoms reported at a single time point refers to women with a SRQ-20 score ≥9 for a single time point only; high episodic depressive symptoms refers to women with a SRQ-20 score ≥9 at 2 or more time points, but not continuously; and chronicity refers to women with a SRQ-20 score ≥9 at 2 or more consecutive time points.

## Discussion

This study of a rural disadvantaged population in Pakistan employed repeated measures of maternal depressive symptoms. The results show 69.5% (N = 838) of children were cared for by mothers who experienced high maternal depressive symptoms during the first 24 months postpartum and 35.5% (N = 428) of these mothers, experienced chronicity of high depressive symptoms.

The findings demonstrated that chronic high depressive symptoms in mothers were not associated with early child cognitive, language, and motor development at 24 months of age after adjusting for other risk factors in the child’s immediate caregiving environment (including caregiver-child interactions, and home environment, household SES, and child height-for-age). However, chronically elevated maternal depressive symptoms were significantly negatively associated with child socio-emotional development and this association was maintained after adjusting for the other risks.

Previous studies have reported postpartum depression was associated with deficits in children’s socio-emotional development (36). The early years are a sensitive period for children’s development, influenced by the mother’s ability to respond to her child’s emotional cues (37). If a mother has reduced emotional availability as a result of depression, she may not have the disposition to interact and respond appropriately to her child’s developmental needs. Maternal emotional disposition, sociability, and assertiveness have been found to predict a higher level of children’s social-emotional development (38), while postnatal depression is associated with low levels of children’s social and emotional development (39) increasing risks for hyperactivity (40), internalizing problems (41), and offspring depression (42).

Only a few studies have previously measured the chronicity of maternal depressive symptoms and associations with child outcomes. Children of mothers with persistent and severe depression are at an increased risk for behavioral problems by 3.5 years of age, and lower mathematics grades and risk of depression during adolescence compared with children whose mothers had normal levels of depressive symptoms below the threshold (43). Dahlen found recurrent episodes of maternal depression in multiple periods of the child life had impacts on poorer mother-infant interactions, reductions in children’s reading scores, less-developed interpersonal skills, and more externalizing behavioral problems in third grade than children whose mothers were not depressed (44). Chronic or persistent postpartum depression can severely compromise the ability of mothers to provide care and foster secure attachment, even more if the symptoms of depression persists in sensitive periods of children’s development in which the impact on the child’s emotional development is potentially more threatened (45).

To the best of our knowledge, the present study is the first from a LMIC to corroborate the evidence from high-income countries on chronicity of high maternal depressive symptoms and early socio-emotional development. However, significant associations between chronic high depressive symptoms and child cognitive, language, or motor development were not found, and for which other risk factors (e.g., early learning opportunities) in the environment likely contribute more significantly. This emphasizes the need for comprehensive intervention approaches that target the needs of the child and the caregiver in contexts of complex disadvantage with exposure to multiple risks. Our findings may be generalizable to similarly disadvantaged populations and contexts with high risk of maternal depressive symptoms. However, caution is warranted given challenges that may limit comparisons. A key issue in the literature is the wide variation in classification of chronicity of maternal depression and its measurement. This variation can make comparisons between studies challenging. However, in general, it can be concluded that severity and persistence of maternal depressive symptoms are associated with poorer early child development outcomes.

The study findings have implications for both programs and research. A program intending to promote and protect early childhood development should consider effective interventions that meet the needs of the child (e.g., support for early development and learning) and care for the primary caregivers for young children, especially the mental health of mothers who are typically the primary caregivers. Training of health professionals on mental health, particularly community health workers, could help to prevent complications and address mental health topics with women. Interventions to promote mental health implemented by lay workers or community health workers in LMICs have been found to be effective as noted in the recent Lancet Commission on Global Mental Health and Sustainable Development (46). Such an approach would require the establishment of a screening system for early identification of caregivers with risks for depression. The treatment of maternal depression is a public health urgency and cannot be neglected in services designed to support young children. With respect to further research, further longitudinal research is needed to elucidate impacts on children’s development in the life course and identify windows of opportunities for early intervention to mitigate negative effects. It is also necessary to have evidence that can distinguish effects on children’s development of chronicity of depression starting in the antenatal and postnatal periods.

The strengths of this study include the large sample, availability of confounding and moderating variables, and the repeated measures of maternal depressive symptoms. This study also has some limitations. While the self-reporting questionnaire SRQ-20 to measure depressive symptoms, a clinical diagnosis of depression was not available. However, a high risk and prevalence of depressive symptoms is reported in studies of maternal depressive symptoms in LMICs which warrants investigation. Further research on a population with clinically diagnosed depression will also be needed to guide targeted intervention work. In addition, information about the antenatal period where depressive symptoms may also affect child outcomes were not collected.

## Conclusions

This study contributes to the growing knowledge about symptoms of chronic maternal depressive symptoms and the impact on children’s socio-emotional development. These results support the need to establish a plan to provide emotional and psychological support for mothers in the sensitive early period of development in the life of the child.

## Data Availability Statement

The datasets generated for this study are available on request to the corresponding author.

## Ethics Statement

The studies involving human participants were reviewed and approved by Aga Khan University Ethical Research Committee. Written informed consent for children to participate in this study was provided by the participants’ legal guardian/next of kin.

## Author Contributions

AY was the PI of the PEDS trial and conceptualized the present analysis and contributed to the drafting of the manuscript. CO analyzed the data and drafted the manuscript. MR reviewed a draft of the manuscript.

## Conflict of Interest

The authors declare that the research was conducted in the absence of any commercial or financial relationships that could be construed as a potential conflict of interest.
